# From atoms to bits: Resource mobilization of non-digital, hybrid, and digital cleantech startups

**DOI:** 10.1016/j.heliyon.2023.e23697

**Published:** 2023-12-14

**Authors:** Susanne Kurowski

**Affiliations:** Technical University of Munich, TUM Campus for Biotechnology and Sustainability, Professorship of Innovation and Technology Management, Am Essigberg 3, 94315 Straubing, Germany

**Keywords:** Cleantech startup, Resource mobilization, Digital entrepreneurship

## Abstract

Climate change is one of the most critical challenges to tackle in this century, where innovations developed and commercialized by cleantech startups are crucial contributors to achieve emission reduction targets. Entrepreneurship scholars have long presumed that resource mobilization is essential for startups to transit successfully through the conception and commercialization life cycle stages. Yet, we have a limited understanding of how resource mobilization varies across the three startups types of non-digital, hybrid – an intermediate type of non-digital and digital startups –, and digital cleantech startups. Drawing on insights from 16 semi-structured interviews with startups, investors, and industry experts in the U.S. cleantech industry, as well as secondary data, this study provides a novel framework that identifies the resource mobilization approaches of cleantech startups disentangled by the three startup types. The findings indicate that non-digital cleantech startups face the most severe resource mobilization challenges, followed by hybrid and digital cleantech startups, respectively. The study contributes to the literature on resource mobilization of cleantech startups and digital entrepreneurship. It also outlines implications for startups and venture capital investors as practitioners as well as for policymakers.

## Highlights

• A novel typology distinguishes non-digital, hybrid, and digital cleantech startups.
• Qualitative analysis is based on 16 semi-structured interviews and secondary data.
• Non-digital cleantech startups face the hardest resource mobilization challenges.
• Policies should be directed toward supporting non-digital cleantech startups.

## 1 Introduction

Mitigating climate change poses one of the most pressing challenges in this century to prevent human-induced temperature changes and associated environmental degradation. To limit severe consequences, the goal of the Paris Agreement is to restrict temperature rise to well below 2 °C above the pre-industrial level, which implies reaching net-zero emissions by 2050 [1]. It is widely recognized that this target is only achievable with the rapid advancement of clean energy technology (cleantech) innovations [2]. Cleantech startups play a pivotal role in developing and commercializing these innovations, thereby generating both economic and environmental value [3]. However, cleantech startups face significant challenges, particularly in mobilizing the necessary resources [4,5]. The inherent liabilities of newness and smallness exacerbate these resource mobilization difficulties for startups [6].

Recent research has found that cleantech startups face additional, sector-specific challenges, while mainly focusing on financial resource mobilization. One stream of studies underscores public support in terms of grants that are necessary for cleantech startups to bridge the long and costly research and development (R&D) time [[7], [8], [9]]. Another stream of studies has investigated the challenges of mobilizing financial resources from venture capital (VC) investors. The literature points to product complexity, low expectations regarding the return on investment, and poor cleantech startup performance in the past that limit the investment appetite of VC investors [[10], [11], [12]]. In these prior studies, entrepreneurial resource mobilization is implicitly linked to life cycle trajectories in the conception and commercialization life cycle stages. Indeed, grants assist cleantech startups in R&D endeavors in the early conception stage, and VC helps cleantech startups pursue commercialization endeavors in the commercialization stage. Interestingly, Howell (2017) shows that grants are more useful for hardware than for software cleantech startups in raising follow-up VC [8]. Further, Gaddy et al. (2017) find that the underperformance of cleantech VC investments is largely driven by deep tech innovations that include material, chemical, and hardware innovations [12]. These insights hint at the possibility of distinct resource mobilization approaches between non-digital and digital cleantech startups during the conception and commercialization stages. Apart from grants and VC investments targeted in previous studies, the progress of cleantech startups in the conception and commercialization stages is also shaped by additional resources, such as partnerships with research institutions and corporations [10,11]. However, within the existing literature on cleantech startups, the resource mobilization approaches beyond financial aspects have not been adequately integrated into the differentiation between non-digital and digital startups.

The differentiation between non-digital and digital startups has already been studied in the digital entrepreneurship literature. Recent digital entrepreneurship studies have begun to compare non-digital and digital startups in terms of business model evolution, strategic patterns, and entrepreneurial orientation [13,14]. However, the critical task of startups to mobilize resources in the conception and commercialization life cycle stages has remained an understudied aspect in the context of non-digital and digital startup comparisons.

Beyond the non-digital/digital startup comparison, a third, hybrid startup type has emerged. The hybrid type has already been associated with smart products and described conceptually in the information systems literature [15,16]. Surprisingly, the literature on digital entrepreneurship has not sufficiently addressed this hybrid startup category.

While there is a growing body of research that has delved into the interplay between digital capabilities and sustainable performance of firms [17,18], there remains a gap in our comprehension of how resource mobilization approaches differ between firms with different product digitization categories. Specifically, this gap extends to non-digital, the intermediate hybrid category that combines non-digital and digital elements, and digital cleantech startups. Consequently, the research question guiding this study is as follows: *How do non-digital, hybrid, and digital cleantech startups mobilize resources in the life cycle stages of conception and commercialization?*

To address the research gaps mentioned above, this study constructs a novel resource mobilization framework for non-digital, hybrid, and digital cleantech startups. Based on the analysis of 16 semi-structured interviews and secondary data, the findings reveal that resource mobilization patterns differ across the startup types of non-digital, hybrid, and digital startups. Specifically, non-digital startups face the most severe resource mobilization challenges, followed by hybrid and digital startups, respectively. This study makes three main contributions. *First*, the resource mobilization framework extends its scope beyond financial resources and illuminates how cleantech startups across the three categories – non-digital, hybrid, and digital – mobilize resources during the critical life cycle stages of conception and commercialization. *Second*, this study contributes to the literature on digital entrepreneurship by integrating resource mobilization into non-digital/digital startup comparisons. In the realm of digital entrepreneurship, it further explores the fundamental assumption that digital products have distinct characteristics, which shape entrepreneurial processes and outcomes. *Third*, this study also extends the established distinction between non-digital and digital startups, and introduces a third type of hybrid startups.

The remaining paper is organized as follows: Section 2 presents the conceptual basis related to entrepreneurial resource mobilization in the conception and commercialization life cycle stages, as well as the startup typology of non-digital, hybrid, and digital startups. After describing the qualitative research methods in section 3, the results in section 4 are organized in line with a novel resource mobilization framework of non-digital, hybrid, and digital startups. In section 5, the results are discussed and theoretical, managerial, and policy implications are outlined. Section 6 draws a conclusion and further highlights limitations and future research opportunities.

## 2 Conceptual basis

This chapter presents the conceptual basis related to entrepreneurial resource mobilization in the conception and commercialization life cycle stages, as well as a typology of digital, hybrid and non-digital startups.

### 2.1 Entrepreneurial resource mobilization in the conception and commercialization stages

A startup is a temporary and innovative organization that delivers a new product, facing a high-growth potential under conditions of extreme uncertainty [22]. Technology startups differ from mainstream startups by pursuing opportunities through technical innovations [23]. Furthermore, VC-backing of startups points to the startups’ aim to liquidate the assets through a successful exit [24]. As a consequence, VC-backed technology startups have different resource mobilization approaches and life cycle dynamics than mainstream startups without VC-backing [5,25]. This makes a scope limitation necessary. In this study, the scope is limited to VC-backed technology startups.

Startups are nascent organizations and typically have limited initial resource endowments. Consequently, resource mobilization is a central task for startups and encompasses processes by which startups assemble resources to exploit opportunities [4,26]. Resources are commonly grouped into four categories: financial, social, human, and other resources [4]. While financial resources refer to monetary assets [27], social resources relate to interorganizational ties through which non-monetary resources are obtained [28,29]. Human resources include capabilities, knowledge, and skills residing in and utilized by individuals related to the startup [30]. For VC-backed technology startups, other resources commonly include tangible and intangible technological resources [16,31] and manufacturing resources [29,32]. Leveraging these resources is a crucial and challenging endeavor for startups to transit successfully through stages in the life cycle [4,5].

Organizational life cycle theory conceptualizes a staged life cycle, where organizations evolve predictably to achieve organizational growth [33]. As regards resource mobilization, this study focuses on the first two life cycle stages – conception and commercialization – of VC-backed technology startups [5]. By focusing on these two early stages, this study explores the specific resource mobilization approaches encountered during the critical early stages of the startup's life cycle, where resource mobilization hurdles are typically the highest due to the liabilities of newness and smallness [6]. Specifically, the conception stage centers on R&D, and the commercialization stage comprises further technological development, including the identification and resolution of technical problems, technology demonstration, and market introduction [5].

Empirical research has confirmed the role of the four resource types for VC-backed technology startups to achieve progress in transiting through the life cycle stages. Regarding *financial resources*, empirical research has provided evidence that accelerators [34], government financial support, such as grants [9], as well as impact investors [35] enable startups to progress in the conception stage. Moreover, empirical research has shown that mobilizing financial resources, particularly from traditional and corporate VC investors, enables startups to advance through the commercialization stage toward the growth stage [27]. *Social resources*, in the conception stage, occur in terms of technology alliances with research institutes [36]. Furthermore, incubators are important in the conception stage as they assist startups with space, administrative services, legal advice, networking opportunities, and potential access to financial resources [37]. In the commercialization stage, social resources in the form of ties with corporates and other startups enhance the venture's performance [38,39]. When considering *human resources*, empirical research points to the importance of the technical knowledge and skills of individuals in the conception stage [40,41]. In the commercialization stage, business-related knowledge and skills improve venture performance [42]. Apart from financial, social, and human resources, the mobilization of *other resources* – specifically technological and manufacturing resources – is crucial. Regarding technological resources, scholars have mainly considered patents, which indicate the invention output of R&D activities in the conception stage [7]. Other empirical studies have shown that patents also function as quality signals to mobilize financial resources in the commercialization stage [43,44]. Finally, research has demonstrated that manufacturing resources are critical in the later life cycle stage of commercialization [29,32].

Taken together, the mobilization of resources, encompassing financial, social, human, and other resources, holds significant relevance for startups as they navigate the life cycle stages of conception and commercialization. Within each of these resource categories, prior research has examined the nuances associated with resource providers, such as accelerators and VC investors, as well as various resource types, such as technical-related knowledge and skills and business-related knowledge and skills. However, there is an important gap in the literature, namely, the lack of a comprehensive integration that distinguishes between non-digital, hybrid, and digital startups within the resource mobilization context.

### 2.2 The typology of non-digital, hybrid, and digital startups

Researchers from the field of entrepreneurship have called for a separate examination of non-digital and digital startups. The direct comparison of non-digital and digital startups has been previously assessed only to a limited extent, where two studies are remarkable. While König et al. (2019) compare business model evolution patterns [14], Kollmann et al. (2021) compare strategic cooperation patterns and the entrepreneurial orientation of non-digital and digital startups [13]. This suggested separation of non-digital and digital startups is integrated into the startup typology in this study. Furthermore, there is evidence from the information systems literature that the previous two startup types converge into a third type. This convergence of the non-digital and digital dimensions is evident in firms that develop and market smart products [16,19,[20], [21]]. This makes the introduction of a third category, termed hybrid startups, relevant. The entrepreneurship literature has not referred to hybrid startups in sufficient detail. Overall, this study takes a product-centric perspective to relate the startup type, i.e., non-digital, hybrid, and digital startups, to the product innovation type, i.e., non-digital, hybrid, and digital products [15,45].

*Digital startups* are characterized by digital product innovations that contain software-enabled product components [45,46]. Accordingly, software enables and manifests in digital product innovation and is an integral part of the nature of products. In essence, digital startups develop and commercialize digital product innovations as the output of entrepreneurial operations. Digital product innovation contains digital product components only.

In contrast, *non-digital startups* are characterized by non-digital product innovations, where the product contains physical components only. Accordingly, software is not manifested as an outcome in non-digital product innovation, but it can be relevant in supporting value-creation activities [13].

In addition to digital and non-digital startups, a third startup type has emerged that combines the characteristics of the previously described two types. The hybridity manifests in product innovations as the combination of digital and physical components to create new products. Hybrid product innovations thus reflect the convergence of the digital and non-digital spheres [15,16]. Accordingly, *hybrid startups* are characterized by hybrid product innovations as the output of entrepreneurial operations.

## 3 Methods

The methods chapter begins by addressing data sampling in the context of selecting interview participants. The qualitative data collection process is then explained before moving on to an explanation of the data analysis, with a particular focus on the coding methodology employed.

### 3.1 Data sampling

The research design involves a qualitative approach, which intends to gain deep insights by relating the typology of non-digital, hybrid, and digital cleantech startups to specific resource mobilization approaches. To gain comprehensive information, a combination of quota and knowledgeable sampling of stakeholders in the U.S. cleantech industry was employed. Quota sampling was used to fill in participants in key categories [47]. In this sense, the number of participants was balanced between the two main categories of startups and investors. In addition to startups, investors have a deep knowledge of the startups' resource mobilization approaches. Indeed, investors need to understand the startups' resource mobilization approaches because it allows them to assess the startups' potential for growth and success. Within each category, it was attempted to cover different perspectives so that agnostic and cleantech-focused investors, as well as startups from various cleantech sub-sectors and types were included. Additionally, industry experts were included as a third category because they hold deep knowledge about the cleantech industry. Industry experts offer an objective viewpoint that complements the perspectives of cleantech startups and VC investors. Across all categories, the participants include key knowledgeable people because they have in-depth knowledge of specialized issues [47]. Key knowledgeables in the three participant categories were defined as follows: Participants of the category investors need to have made at least one investment in a cleantech startup and have at least five years of investing experience. Participants of the category cleantech startups need to have received at least one VC investment and have at least five years of work experience in a cleantech startup. The experts need to have at least five years of work experience in the cleantech industry. All interview participants were approached via email, containing a brief personal introduction and the purpose of the interview. Table 1 shows the classification and specification of the participants’ associated firm, the position of the participants, and the number of venture investments made by an investor or the number of investors who made investments in a startup as well as the recording time of the interview in minutes.

**Table 1:** Overview of interview participants.

Acronym^a^	Classification (specification)	Position of the participant	Venture investments^b^	Recording time (in min.)
I1	Investor (agnostic)	Founding Partner	I: 201	56
I2	Investor (agnostic)	Partner	I: 96	30
I3	Investor (agnostic)	Managing Partner	I: 350	28
I4	Investor (cleantech-focused)	Partner	I: 24	29
I5	Investor (cleantech-focused)	Partner	I: 6	30
I6	Investor (cleantech-focused)	Managing Director	I: 36	47
I7	Investor (cleantech-focused)	Director	I: 61	50
S1	Non-digital startup (energy efficiency)	Founder; CEO	S: 6	31
S2	Digital startup (energy efficiency)	Co-founder; CEO	S: 8	26
S3	Non-digital startup (energy efficiency)	Founder; CEO	S: 13	29
S4	Hybrid startup (energy storage)	Vice President	S: 3	37
S5	Non-digital startup (geothermal)	CEO	S: 15	41
S6	Hybrid startup (smart grid)	Founder; CEO	S: 1	30
E1	Expert (energy consulting)	Associate Director	E: NA	34
E2	Expert (startup consulting)	CEO	E: NA	22
E3	Expert (sustainability consulting)	Founder	E: NA	28
				Total: 548

a I = investor; S = startup; E = expert.

b I = Number of investments made by an investor I; S = number of investors who made investments in a startup S; NA = not assigned to expert E (data retrieved from Crunchbase in August 2021).

### 3.2 Data collection

The data was collected through semi-structured interviews, which follow a pre-formulated structure while allowing interviewers to add questions and participants to reveal additional insights as they appear during the conversation [48]. A total of 16 semi-structured interviews were conducted between June and August 2021. Three core themes were covered: product characteristics, startup characteristics, and resource mobilization of non-digital, hybrid, and digital cleantech startups. Accordingly, the three main preformulated questions are: (1) What are the specific and different characteristics of the products of non-digital, hybrid, and digital startups? (2) What are the specific and different characteristics of non-digital, hybrid, and digital startups? (3) How does the mobilization of financial, social, human and other resources differ between non-digital, hybrid and digital startups?

All interviews were held via Zoom. All interviews were recorded. The interview recordings lasted between 22 and 56 min. In total, 548 min (approximately 9 h) of audio recording were obtained. The audio memos were transcribed verbatim. The transcripts were triangulated with secondary data, such as publicly available information on company websites and LinkedIn posts of the companies and participants, to obtain further information on the retrospective and real-time accounts of those participants experiencing the resource mobilization phenomenon [49].

### 3.3 Data analysis

For the data analysis, a combination of the deductive and inductive approaches was applied [50]. For the coding frame, the statements were initially assigned to the startup type they were referring to, i.e., to non-digital, hybrid, and digital startups. Then, six main categories were deductively set up. In line with Clough et al. (2019), resource mobilization based on the four resource types was specified [4]. Accordingly, the six main categories were: (1) product characteristics, (2) startup characteristics, (3) financial resource mobilization, (4) social resource mobilization, (5) human resource mobilization, and (6) mobilization of other resources. In two rounds of transcript examination, sub-categories for each main category were inductively established. Simultaneously, a research assistant also established sub-categories inductively from the transcripts. The sub-categories were compared and discussed, which finally resulted in two to six sub-categories for each main category. After establishing the coding frame, the author and the research assistant coded the data. In case of discrepancies, the transcribed text segments were carefully reviewed and the final codes were assigned. All interviews were coded manually using the software MAXQDA version 2020. Finally, a life cycle stage was assigned, i.e., conception or commercialization, to each sub-category [5]. Table 2 summarizes the arrangement of the three core themes, six main categories, and associated sub-categories, as well as the assignment of the life cycle stage to the three types of startups.

**Table 2:**
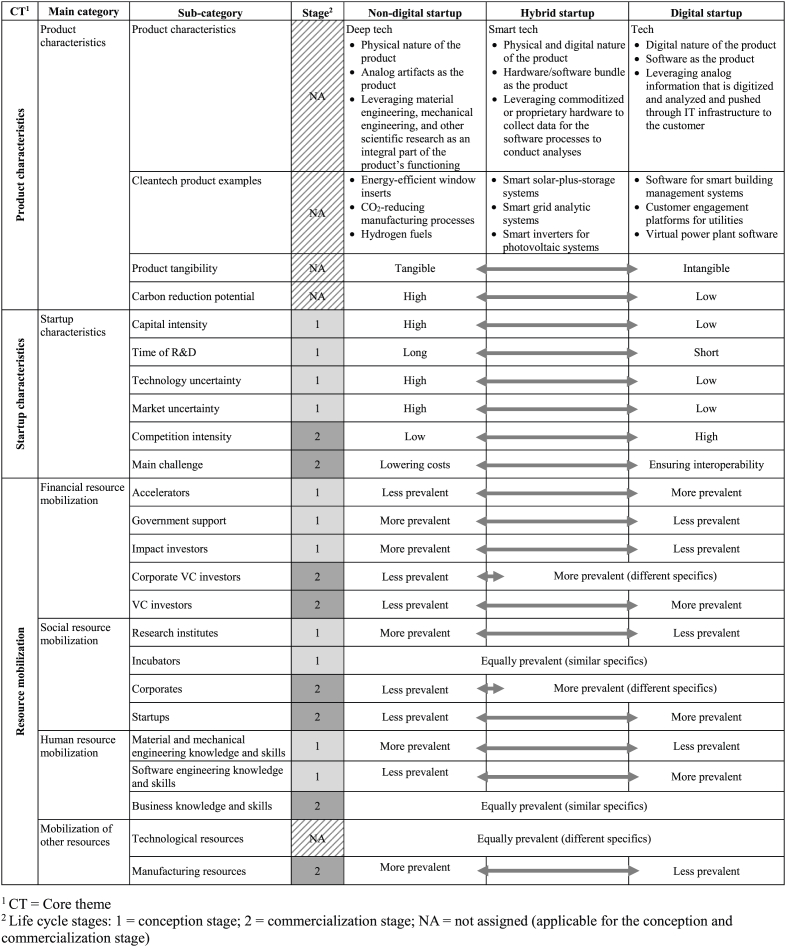
Framework of product characteristics, startup characteristics, and resource mobilization of non-digital, hybrid, and digital startups.

## 4 Results

Table 2 also summarizes the results. The results are presented sequentially for each startup type. Within each startup type, the results are then structured according to the six main categories: (1) product characteristics, (2) startup characteristics, (3) financial resource mobilization, (4) social resource mobilization, (5) human resource mobilization, and (6) mobilization of other resources.

### 4.1 Non-digital startups

#### 4.1.1 Product characteristics of non-digital startups

The product characteristics of non-digital startups are related to deep tech, which stresses the engineering and scientific aspects of non-digital products. The interview participants highlighted the physical nature and analog artifacts that compose the product. Specifically, they emphasized that material and mechanical engineering and other scientific research are integral parts of the product's functioning. Examples of non-digital product innovation in the cleantech industry are energy-efficient window inserts, CO_2_-reducing manufacturing processes (for instance, CO_2_-reducing steel and cement production processes), and hydrogen fuels. Overall, the products are entirely tangible. Interestingly, the interview participants classified the carbon reduction potential of non-digital products in the cleantech industry as high, which depends on technological specifics and product quantity sold.

#### 4.1.2 Startup characteristics of non-digital startups

Regarding startup characteristics, the interview participants emphasized the high capital intensity of non-digital startups in the conception stage. In addition, they assessed the time of R&D as long. Both the high capital intensity and the long period of R&D contribute to an extremely long and deep valley of death. In the conception stage, the interview participants characterized the technology uncertainty as high. In addition, the market uncertainty was evaluated as high because of the absence of a market in the future in the extreme case.

In the commercialization stage, the competition intensity of non-digital startups was characterized as low due to the scientific complexity of the non-digital products, which makes it challenging to imitate products. The main challenge for non-digital startups in the commercialization stage is to lower production costs to sell the product at lower prices. For example, the chief executive officer (CEO) of a non-digital geothermal startup noted that it is important to have “more efficient drilling tools that lower the costs” (S5) in the commercialization stage.

#### 4.1.3 Financial resource mobilization of non-digital startups

Mobilizing significant amounts of financial resources is important but challenging for non-digital startups. Regarding financial resource providers in the conception stage, accelerators are less prevalent for non-digital startups than for the other two startup types. Although accelerators can be beneficial for non-digital startups to secure initial pre-seed and seed funding, it is often difficult for non-digital startups to get accepted. The founder and CEO of a non-digital startup described the experience in the accelerator as follows: “We were literally the only hardware company [in the accelerator]. We were like a zoo exhibit” (S1).

Apart from accelerators in the conception stage, government financial support and impact investors are more prevalent for non-digital startups than for hybrid and digital startups. Mobilizing financial resources through government support, especially grants and loans, is prevalent for non-digital startups. According to the interview participants, government support is crucial to fund non-digital startups that, on the one hand, have a high carbon reduction potential and, on the other hand, suffer from the disregard of other financial resource providers. Overall, an important advantage of government financial support is that startups can buy some time to continue working on their product innovations without being pushed into the timelines of private investors: “At the early R&D stage, scientists and engineers may suffer from the distraction of interacting with commercial VC investors. […] Once a company takes VC financing, the timeline starts becoming specific. A precise timeline does not necessarily fit with the search and R&D for a breakthrough in a technology” (I4).

In the conception stage, impact investors also play a critical role for non-digital startups. Impact investors typically invest before VC investors participate. The interview participants revealed that impact investors structure funds either by pooling the money from philanthropic or individual donors or by relying on large funds of corporate foundations. Impact investors have a “heavy climate lens” (I6) and focus on the decarbonization potential of the product innovation, which is predominately given by non-digital startups. Their expectation regarding the return on investment is positive but not as high as that of VC investors. Funds of impact investors usually have a longer investment time horizon, potentially including evergreen funds, and often function on a revolving basis. Moreover, impact investors often have internal technical experts or access to expert knowledge via partnerships with universities and research institutes. They can, therefore, conduct due diligence on non-digital startups competently.

In the commercialization stage, corporate VC investors are less prevalent for non-digital startups. Corporate VC investors can potentially be interesting partners for non-digital startups to mobilize financial and other resources. However, interest conflicts related to intellectual property often emerge. For example, a non-digital startup founder mentioned that a “strategic partner could understand the technical aspects. […] However, there was ultimately divergence on intellectual property considerations” (S1). So the deal was not closed.

Concerning VC investors, the interview participants assessed them as less prevalent for non-digital startups. This was due to the extremely long and deep valley of death, which is “not compatible with venture-style returns” (I5). VC investors also stated that material and mechanical engineering science is very complex, so they are not able to conduct due diligence on non-digital startups competently. All non-digital startup founders and CEOs agreed that it was difficult to raise money from traditional VC investors. However, the interviews also stressed that traditional VC investors were increasingly setting up climate-oriented funds, intending to make more investments in non-digital cleantech startups acknowledging their high carbon reduction potential.

#### 4.1.4 Social resource mobilization of non-digital startups

In the conception stage of non-digital startups, research institutes are prevalent. They are significant partners for non-digital startups to build R&D alliances to access physical resources, such as facilities and laboratories, as well as human resources, such as the technical knowledge and skills of employees at the research institute. 10.13039/100014337Furthermore, in the conception stage, incubators are vital social resource providers, enabling startups to access space, administrative support, and new personal networks.

In the commercialization stage, corporates overall play a less decisive role for non-digital startups to mobilize resources. As an exception, one non-digital startup founder described that they had formed a development alliance with a corporate for non-recurring engineering.

Alliances with other startups are also less prevalent and have an informal character. The participants indicated that such alliances only include the exchange of business-related knowledge. These kinds of relations are collegial and can contain knowledge exchange about “fund-raising plans” (S1) and “mistakes of others” (S5).

#### 4.1.5 Human resource mobilization of non-digital startups

For non-digital startups, it is relevant to mobilize material and mechanical engineering knowledge and skills to conceptualize and build prototypes in the conception stage. In contrast, the interviews revealed that software engineering knowledge and skills are less prevalent in the conception stage due to the absence of digital product development. In the commercialization stage, the focus shifts toward business knowledge and skills, especially in terms of firm-level collaborations and scaling endeavors, which are necessary for non-digital startups to commercialize their products.

#### 4.1.6 Mobilization of other resources of non-digital startups

Throughout the conception and commercialization stages, technological resource mobilization is important for non-digital startups. It includes access to facilities and lab equipment in the tangible dimension and patents in the intangible dimension.

Furthermore, mobilizing manufacturing resources is vital for non-digital startups in the commercialization stage. The interview participants revealed two options for non-digital startups to mobilize manufacturing resources: building or contracting. As it requires “tons of capital expenditure to build plants” (I5), the building option is rarely chosen by non-digital startups. Accordingly, the interview participants emphasized that contracting is a more suitable option for non-digital startups.

### 4.2 Hybrid startups

#### 4.2.1 Product characteristics of hybrid startups

The characteristics of products of hybrid startups are related to smart tech. The product nature is characterized as both physical and digital. The interview participants described a hybrid product as a hardware/software bundle. Specifically, the software processes data, which were collected by commoditized or proprietary hardware, to conduct analyses. These data analyses often include predictive models. The hardware could potentially function without the software, but the value of the hybrid product is created by tying the software to the hardware. Examples of hybrid products in the cleantech industry include smart solar-plus-storage systems, smart grid analytic systems, and smart inverters for photovoltaic systems. As the hardware components of the product are tangible and the software components are intangible, the interview participants assessed the degree of tangibility as moderate, positioned between the extremes of tangible and intangible. The interview participants indicated that the carbon reduction potential of hybrid products is moderately high, i.e., lower than for non-digital cleantech products but higher than for digital cleantech products.

#### 4.2.2 Startup characteristics of hybrid startups

The capital intensity of hybrid startups in the conception stage was assessed as moderately high. While software-related product development is less capital intensive, hardware-related product development is more capital intensive, especially in the case of proprietary hardware as compared to commoditized hardware. According to the interview participants, the time of R&D is moderately long. The interview participants revealed that the user experience is compelling for hybrid startups in the conception stage, where overall shorter iterations of development and testing lead to a shorter time of R&D compared to non-digital startups. Regarding technological and market uncertainty, both were evaluated as moderately high but lower than for non-digital startups.

In the commercialization stage, the competition was characterized as moderately intense and higher than for non-digital startups. The interview participants stated that easy access to and cost drops of commoditized hardware, particularly sensors, pave the way for more startups to develop and commercialize hybrid products, which increases the competition intensity. The challenge for hybrid startups in the commercialization stage centers on lowering costs, which is also the focal commercialization challenge of non-digital startups. They must also ensure interoperability, which accounts for the main commercialization challenge of digital startups. In particular, industry experts noted that interoperability with other smart (or hybrid) products and the existing information technology (IT) infrastructure poses a crucial challenge for hybrid startups in the commercialization stage.

#### 4.2.3 Financial resource mobilization of hybrid startups

Regarding financial resource providers in the conception stage, accelerators are moderately prevalent. Government financial support for hybrid startups was also assessed as moderately prevalent. The interview participants indicated that government financial support is not as vital and prevalent as for non-digital startups.

Impact investors in the conception stage were evaluated as moderately prevalent. Regarding the double-bottom line of impact investors consisting of economic and environmental values, all hybrid startups noted that they were asked to quantify the environmental impact in terms of a “carbon analysis to support the product” (S4).

Interestingly, the interview participants stated that corporate VC investors are more prevalent for hybrid startups than for non-digital startups. Hybrid startups identified hardware manufacturers and telecommunication and IT firms as promising corporate VC investors. Corporate VC investors do not only offer financial resources but also other resources. While hardware manufacturers provide manufacturing resources, telecommunication and IT firms provide technological resources, such as IT infrastructure in terms of, for example, cloud storage and servers.

Traditional VC investors are moderately prevalent in the commercialization stage of hybrid startups. The investors perceived that hybrid startups achieve higher returns on investment than non-digital startups. Hybrid startups noted that they emphasized the software components and deemphasized the hardware component when pitching to agnostic VC investors, as VC investors generally prefer investments in digital startups.

#### 4.2.4 Social resource mobilization of hybrid startups

Alliances with research institutes are less prevalent for hybrid startups than for non-digital startups. Indeed, hybrid startups are not as dependent on resource transfer from research institutes because facilities, engineering, and scientific knowledge and skills are not as pertinent. Similar to non-digital startups, incubators are important social resource providers for hybrid startups in terms of the provision of space, administrative support, and networking opportunities.

In the commercialization stage, mobilizing resources from corporates is more prevalent for hybrid than for non-digital startups. Indeed, hybrid startups often establish ties with corporates to contract manufacturing resources. Known hardware product components are assembled by manufacturing corporates. These corporates can be prior corporate VC investors.

Startup alliances have a mixed purpose for hybrid startups. On the one hand, informal exchange of business-related information, such as in the case of non-digital startups, is prevalent. On the other hand, they also pursue value co-creation endeavors, such as in the case of digital startups. Therefore, mobilizing resources from other startups is assessed as moderately prevalent.

#### 4.2.5 Human resource mobilization of hybrid startups

In the conception stage, hybrid startups mobilize material and mechanical, as well as software engineering knowledge and skills. Both kinds of knowledge and skills are moderately prevalent. On the one hand, material and mechanical engineering knowledge and skills are important for hardware engineering. On the other hand, software engineering knowledge and skills are important for software engineering. Thus, in the commercialization stage, hybrid startups need to mobilize collaboration and scaling knowledge and skills as part of business knowledge and skills.

#### 4.2.6 Mobilization of other resources of hybrid startups

Across both life cycle stages, hybrid startups need to mobilize technological resources. According to the interview participants, leveraging commoditized or proprietary hardware is essential and can occur, as described above, through corporate alliances and prior corporate VC investor affiliations. Moreover, the interviews revealed that data and IT infrastructure account for vital technological resources, which become increasingly important with more digital components embedded in hybrid products. Technological resources can also be the ones used by non-digital startups, i.e., facilities, lab equipment, and patents.

The mobilization of manufacturing resources in the commercialization stage is less prevalent than for non-digital startups, and it presents less of a challenge. Often, the hardware (e.g., sensors) is available as a commodity on the market. If contract manufacturers are engaged, they can manufacture the hardware required, as it often consists of existing product components.

### 4.3 Digital startups

#### 4.3.1 Product characteristics of digital startups

The product characteristics of digital startups are related to tech, which indicates the digital nature of the software product. Specifically, the analog information is digitized and analyzed by the software. Results of (predictive) data analytics are then pushed to the customer using an IT infrastructure. Digital cleantech products comprise, for example, software for smart building management systems or customer engagement platforms for utilities, which provide detailed home energy reports to customers, or software for virtual power plants. Overall, digital products are characterized as purely intangible. Interestingly, the carbon reduction potential of digital products was assessed as low. According to the participants, digital products induce environmental efficiency improvements of existing products or infrastructure. Thus, the carbon reduction potential of digital products depends on the environmental efficiency improvement potential of an existing product or infrastructure and the product quantity (e.g., licenses) sold.

#### 4.3.2 Startup characteristics of digital startups

In the conception stage of digital startups, the interview participants assessed the capital intensity as low and the time for R&D as short. Furthermore, both the technology and market uncertainty of digital startups in the conception stage were estimated as low. In the commercialization stage, the competition intensity was evaluated as high. The interview participants indicated that low capital intensity and widely available software engineering knowledge and skills facilitate the imitation of digital products and thereby increase the competition intensity among digital startups. The interviews revealed that the main challenge of digital startups in the commercialization stage centers on ensuring interoperability. As digital products in the cleantech industry improve the environmental efficiency of existing products or infrastructure, digital products need to be interoperable.

#### 4.3.3 Financial resource mobilization of digital startups

Concerning financial resource providers in the conception stage, accelerators are more prevalent for digital than for non-digital and hybrid startups. The interviews revealed that many accelerators focus on digital startups, enabling higher acceptance rates. In the conception stage, other financial resource providers, such as government financial support and impact investors, were assessed as less prevalent than for non-digital and hybrid startups.

In the commercialization stage, the interview participants revealed that corporate VC investors are similarly prevalent as for hybrid startups and thereby, more prevalent than for non-digital startups. Telecommunication and IT firms and utilities were mentioned most frequently as corporate VC investors for digital startups. These firms are often strategically interested in the digital cleantech products developed and commercialized by the startups.

VC investors in the commercialization stage were assessed as more prevalent for digital than for non-digital and hybrid startups. The interview participants argued that VC investors’ preference for digital startups is linked to the short and shallow valley of death that occurs due to low capital intensity and a shorter R&D time. This leads VC investors to perceive that digital startups achieve higher return of investments than non-digital and hybrid startups. For example, an investor noted: “A typical good performing venture portfolio should achieve a 25 %–30 % return. You can hit that when you invest in pure software companies” (I5). The investors also revealed that path dependence on digital startups as investment targets drives their investment preferences. VC investors have been most experienced with investing in digital startups, and thus, their due diligence competencies are often limited to this startup type. As put by one investor: “For VC investors that do not have access to technical experts who can do due diligence, it is easier to simply look for patterns across software startups” (I4).

#### 4.3.4 Social resource mobilization of digital startups

The interviews revealed that alliances with research institutes in the conception stage are less prevalent for digital startups because they do not require access to physical resources, such as facilities and laboratories, or the technical knowledge and skills of employees at the research institute.

Similar to non-digital and hybrid startups, incubators are vital social resource providers for digital startups in the conception stage to access space, administrative support, and networking opportunities.

In the commercialization stage, ties to corporates are valuable for digital startups. The interview participants revealed that many digital startups are selling to business customers that predominately include telecommunication and IT firms and utilities. These customer alliances are often facilitated by traditional VC investors: “We introduce the customers to our digital startups. I make an introduction to connect them” (I1).

For digital startups, alliances with other startups are more prevalent than for the two other startup types. The interviews revealed that digital startups form alliances with other digital startups for value co-creation. All investors agreed that value co-creation alliances are most beneficial for digital startups as knowledge and skills in the software domain form a common ground for sharing skills and knowledge. Digital startup alliances are often facilitated by VC investors. In this context, an investor elaborated: “We have definitely done cross-pollination of our portfolio companies so that they were able to conduct business around each other” (I2). However, another investor also pointed to competitive frictions when facilitating value co-creation between digital startups: “There may be opportunities for collaboration, but you may run into more issues related to competition as well” (I6).

#### 4.3.5 Human resource mobilization of digital startups

In the conception stage, mechanical and material engineering knowledge and skills are less prevalent as the product does not contain physical components. In the life cycle stage of conception, software engineering knowledge and skills are very prevalent. Overall, the interview participants assessed the availability of software engineering knowledge and skills as high. They argued that software engineering knowledge and skills are not sector-specific and, thus, universally available. As an investor argued: “You do not need your backend developer to understand greenhouse gas emissions” (I6). As in the case of non-digital and hybrid startups, the interview participants revealed that business skills and knowledge in the commercialization stage universally refer to collaboration and scaling.

#### 4.3.6 Mobilization of other resources of digital startups

Digital startups also need to mobilize other resources. In this regard, mobilizing technological resources requires digital startups to access (real-time) data and IT infrastructure, including, for example, cloud storage and servers. The mobilization of manufacturing resources in the commercialization stage is not prevalent because no physical product components are embedded in the product.

## 5 Discussion

This chapter engages in a detailed discussion of the findings, accompanied by the presentation of theoretical, managerial, and policy implications.

### 5.1 Theoretical contributions

This study provides a novel resource mobilization framework of non-digital, hybrid, and digital cleantech startups in the life cycle stages of conception and commercialization. The findings enable three main contributions.

*First*, this study provides evidence that not only sector-specific challenges, such as product complexity and poor cleantech startup performance in the past, are pertinent to cleantech startups [10,11], but resource mobilization challenges differ within the cleantech sector and can be differentiated by the startup type – non-digital, hybrid, or digital. The results indicate that non-digital startups encounter the most severe resource mobilization challenges, followed by hybrid and digital startups, respectively. Notably, the challenges faced by non-digital startups are closely intertwined with their startup characteristics. These findings align with prior research by Gaddy et al. (2017), which emphasizes the greater attractiveness of digital cleantech startups to VC investors compared to non-digital startups focused on deep tech products [12]. In addition to concentrating on financial resources, this study contributes to the existing body of literature by delving into the distinct facets of social, human, and other resources across the three categories of startups [10,11]. Furthermore, this study unveils the influence of the startup type on the carbon reduction potential of cleantech products, with non-digital products being assessed as having the highest potential, followed by hybrid and digital products, respectively. This linkage between carbon reduction potential and startup types enriches the current literature on the intersection of digitization and sustainable entrepreneurship [51].

*Second*, this study further extends the stream of studies that compare non-digital and digital startups. Recent research in the field of digital entrepreneurship has commenced examining the distinctions between non-digital and digital ventures. König et al. (2019) differentiated between these two types based on the tangibility of the core product and demonstrated that non-digital and digital ventures follow distinct evolutionary business model patterns [14]. Kollmann et al. (2021) differentiated non-digital and digital startups based on industry assignment and found that different combinations of internal and external characteristics spur innovations in non-digital and digital startups [13]. This study takes a product-centric lens on digitization [15,45] and hence, the findings reveal that technological shifts in digitization not only manifest in products, and accordingly, in startup types, but they have consequences for the approaches to entrepreneurial resource mobilization. Thereby, this study responds to recent calls not only to consider digitization as a context, but also to explore the fundamental assumption that digital products have distinct characteristics, which shape entrepreneurial processes and outcomes [52,53].

*Third*, this study introduces a third category of hybrid startups that, from a product-centric perspective, lies between the two extreme categories of non-digital and digital startups. Hybrid startups develop and commercialize smart products. Those products contain both non-digital and digital components and have already been described in the information systems literature conceptually [15,16]. By introducing the hybrid startup type, insights from the information systems literature are transferred to the growing field of digital entrepreneurship [53].

### 5.2 Managerial implications

This study has important practical implications for startups and VC investors. The findings suggest that non-digital startups should be prepared to present the carbon reduction potential of the non-digital product when pitching to financial resource providers. This can be especially relevant when pitching to impact investors [54]. For non-digital startups, mobilizing financial resources from VC investors remains critical but challenging [12]. Therefore, non-digital startups should extensively signal their underlying quality to VC investors, e.g., by demonstrating the quality of the founding team, patents, prior achievements, and affiliations [55]. Another critical issue mentioned during the interviews is that VC investors often lack due diligence capabilities in evaluating the technical specifics of non-digital products. One recommendation is that VC investors should actively engage in networking with research institutes, impact investors, and universities to access these required capabilities for evaluating non-digital products.

Another recommendation for hybrid startups is to focus on corporate VC investors as essential partners for mobilizing financial and other resources, e.g., manufacturing resources [56]. Similar to non-digital startups, they should also be prepared to present the carbon reduction potential of their products to investors.

Concerning digital startups, VC investors should take on an active broker role to connect digital startups with corporates and other digital startups for value co-creation. In addition, digital startups should actively seek these connections with corporates and other digital startups.

### 5.3 Policy implications

The findings drawn from this study have important implications for policymakers. Considering the high carbon reduction potential of non-digital cleantech startups, the policy implications in this section focus on non-digital startups. Specifically, the findings reveal that there is a significant funding gap after the conception stage for non-digital startups. Although it is a controversial topic whether to extend funding beyond the conception stage, public funding could prove to be valuable here. Indeed, pushing non-digital startups toward commercialization is essential because private sector investments largely fail to fund non-digital startups beyond the conception stage, and the societal benefit stemming from the commercialization of non-digital cleantech products is high. Government-funded programs could provide non-digital startups with vital access to financial resources beyond the conception stage, for example, by co-investing with private investors.

Moreover, the interviews revealed that agnostic VC investors lack due diligence competencies for non-digital startups, which is one reason they disregard non-digital startups as investment targets. Government policy could be directed to connect non-digital startups and VC investors with research institutes, impact investors, and universities that can evaluate the technical specifics of the non-digital products. Initiating and empowering these alliances could help guide VC investors’ due diligence efforts toward non-digital startups by mitigating uncertainty related to the technical specifics. Therefore, besides supporting the cleantech startup as the focal unit, expanding support toward the crucial stakeholders in the ecosystem is an important policy recommendation.

## 6 Conclusions, limitations, and future research

This study shows that cleantech startups can be categorized as non-digital, hybrid, and digital, thereby illustrating distinct startup categories spanning from the tangible sphere (“atoms”) to the digital sphere (“bits”). Startups in different categories exhibit distinctive variations in terms of their product characteristics, startup characteristics, and approaches to resource mobilization. This research shows that non-digital cleantech startups encounter the most severe obstacles to mobilizing resources, with hybrid startups following suit, and digital startups facing relatively less pronounced challenges.

Although the focus of the study is to investigate the mobilization of financial, social, human, and other resources across the conception and commercialization stages of different startup types, the findings center on financial resources. On the one hand, this is related to the selected interview participant category of investors that emphasize financial resource mobilization due to their occupation. On the other hand, startups and industry experts also discussed financial resource mobilization extensively during the interviews. While this study highlights financial resource mobilization compared to other types of resources, it also reflects the pronounced role of financial resources for startups [4]. Nevertheless, it would be helpful to conduct further qualitative studies, and include interviews with representatives of corporate VC investors, research institutes, and incubators, to understand the mobilization of other resource types in more detail.

Furthermore, it is important to note that the findings are sector-specific. In particular, the carbon reduction potential and the involvement of impact-driven financial resource providers are specific to the cleantech industry. Therefore, future research could compare entrepreneurial resource mobilization of the three startup types in climate-oriented sectors along with other high-tech sectors, such as financial technology and biotechnology.

In the realm of digital sustainable entrepreneurship, there exists a crucial need to elucidate the relationship between digitization, sustainability, and resource mobilization. Beyond merely contextualizing sustainability within a specific sector, such as the cleantech industry, there is potential value in refining our comprehension of sustainability in terms of both sustainable performance and sustainable value creation [51]. It would be interesting to explore how digitization impacts these aspects in the startup context. Additionally, investigating external factors, such as competition, in the context of resource mobilization approaches of the three startup types presents an intriguing avenue for further research.

This work also provides promising avenues for further quantitative research within the field of environmental entrepreneurship. The findings of this study present a general classification of the environmental impact of products into high, moderate, and low categories for non-digital, hybrid, and digital products, respectively. Although this classification is based on the interviews, it should be noted that different carbon reduction potentials of products may exist within each startup type. Thus, it would be interesting to quantitatively assess the carbon reduction potential of products and further investigate the causal relationship between the environmental impact and entrepreneurial outcomes at the startup level by using surveys or novel tools, such as Crane (www.cranetool.org).

## Ethical declaration

This study adheres to the DGS/BDS ethical codex published in 2017 by the German Sociology Society (Deutsche Gesellschaft für Soziologie, DSG) and the Professional Association of German Sociologists (Berufsverband Deutscher Soziologinnen und Soziologen, BDS) [57]. In accordance with §2 Rights of Participants, informed consent was obtained from all interview participants. Participants were informed about the purpose and methods of the study and their voluntary participation. Participants were also assured that all data remains confidential and that confidentiality remains during data analysis and reporting of results. All electronic recordings were made with the consent of the participants. Access to interview recordings and transcripts was controlled by the author and only guaranteed to the research assistants who assisted during the interviews and supported the coding process. The research assistants were informed about the content of the DGS/BDS ethical codex. This research is integral to the author's Ph.D. thesis.

The methods employed in this study, encompassing ethical considerations, underwent a thorough assessment in collaboration with the author's supervisor at the author's home university (Technical University of Munich) and were approved prior to the initiation of data collection.

## Data availability statement

The data has been used is confidential.

## CRediT authorship contribution statement

**Susanne Kurowski:** Conceptualization, Data curation, Formal analysis, Methodology, Project administration, Software, Writing - original draft, Writing - review & editing.

## Declaration of competing interest

The authors declare the following financial interests/personal relationships which may be considered as potential competing interests:This paper is part of the author's dissertation “Resource Mobilization of Cleantech Startups: Perspectives on Political Ideology and Product Digitization” (see chapter 4; https://mediatum.ub.tum.de/doc/1663872/1663872.pdf).
